# High-throughput screen detects calcium signaling dysfunction in typical sporadic autism spectrum disorder

**DOI:** 10.1038/srep40740

**Published:** 2017-02-01

**Authors:** Galina Schmunk, Rachel L. Nguyen, David L. Ferguson, Kenny Kumar, Ian Parker, J. Jay Gargus

**Affiliations:** 1 Department of Physiology and Biophysics, School of Medicine, University of California, Irvine, California, USA; 2 Center for Autism Research and Translation, University of California, Irvine, California, USA; 3 Department of Neurobiology and Behavior, School of Biological Sciences, University of California, Irvine, California, USA; 4 Division of Human Genetics & Genomics, Department of Pediatrics, School of Medicine, University of California, Irvine, California, USA

## Abstract

Autism spectrum disorder (ASD) is a heterogeneous group of neurodevelopmental disorders without any defined uniting pathophysiology. Ca^2+^ signaling is emerging as a potential node in the genetic architecture of the disorder. We previously reported decreased inositol trisphosphate (IP_3_)-mediated Ca^2+^ release from the endoplasmic reticulum in several rare monogenic syndromes highly comorbid with autism – fragile X and tuberous sclerosis types 1 and 2 syndromes. We now extend those findings to a cohort of subjects with sporadic ASD without any known mutations. We developed and applied a high throughput Fluorometric Imaging Plate Reader (FLIPR) assay to monitor agonist-evoked Ca^2+^ signals in human primary skin fibroblasts. Our results indicate that IP_3_ -mediated Ca^2+^ release from the endoplasmic reticulum in response to activation of purinergic receptors is significantly depressed in subjects with sporadic as well as rare syndromic forms of ASD. We propose that deficits in IP_3_-mediated Ca^2+^ signaling represent a convergent hub function shared across the spectrum of autistic disorders – whether caused by rare highly penetrant mutations or sporadic forms – and holds promise as a biomarker for diagnosis and novel drug discovery.

Autism Spectrum Disorder (ASD) is a common complex polygenic disorder characterized by difficulties in social interaction, communication and restricted, repetitive behaviors. The symptoms and severity vary widely across autistic individuals, complicating diagnosis of this complex spectrum encompassing many phenotypes and co-morbidities, and giving rise to a tragic “diagnostic odyssey” that delays diagnosis, and hence treatment, until the typical mean age of 5yrs^1^. Diagnosis of ASD is made based on questionnaires and behavioral tests, relying on parent observations and comprehensive evaluation by psychologists, pediatricians, psychiatrists, and speech therapists (for a recent review, see ref. 2). The current lack of biomarkers and molecular targets makes diagnosis, study and treatment of ASD a challenging task. Moreover, early diagnosis is critical for optimal intervention^3^,^4^, and accurate diagnosis is crucial in order to exclude other potential conditions which may require different therapies.

Recent advances in genetics have improved our understanding of the pathophysiology of ASD and provided genetic models for studying this condition (for a review, see ref. 5). A handful of monogenic syndromes have been identified, together with over 800 individual genes contributing to susceptibility for autism^6^,^7^,^8^,^9^. These findings indicate that although one highly penetrant mutation is enough to cause ASD^5^,^10^, this is very rare, and the number of potentially contributory genes is too large to be of diagnostic utility. Although highly heritable, the polygenic pattern of ASD inheritance^11^ implies that heterogeneous, weakly penetrant genetic variants – either arising *de novo* or inherited from parents – act in combination with environmental risk factors to cause ASD^12^,^13^. The field has thus begun to migrate from the study of single genes and monogenic disorders, such as fragile X (FXS) and tuberous sclerosis syndromes (TSC), to envisaging how numerous susceptibility factors may converge on a common functional pathway, such as excitation/inhibition^14^,^15^,^16^,^17^, synaptic transmission^18^,^19^,^20^ or Ca^2+^ homeostasis^21^,^22^,^23^,^24^ to exert their deleterious effects. We focus on Ca^2+^ signaling as a compelling potential root defect in the disorder, in light of the growing genetic evidence supporting its role in susceptibility to ASD^21^,^23^,^24^,^25^, and its ubiquitous participation in cellular functions as diverse as neuronal excitability^26^,^27^, neurotransmitter release^28^,^29^, cell secretion^30^,^31^, gene expression, and apoptosis^32^,^33^.

We previously identified depressed inositol trisphosphate (IP_3_)-mediated Ca^2+^ signaling as a shared feature in three distinct monogenic syndromes highly comorbid with ASD – FXS and tuberous sclerosis syndrome type 1 and type 2 (TSC1 and TSC2)^34^. To evoke cytosolic Ca^2+^ liberation through IP_3_ receptor/channels (IP_3_Rs) in the endoplasmic reticulum (ER), we bath applied adenosine triphosphate (ATP) to activate metabotropic purinergic receptors in skin fibroblasts. Responses in fibroblasts obtained from subjects exhibiting each of these monogenic syndromes were depressed as compared with matched controls. These deficits could not be attributed to underfilling of ER Ca^2+^ stores, or to diminished expression of IP_3_R proteins. We then investigated whether they arose through defects in generation of IP_3_ by G-protein coupled receptor (GPCR) activation of phospholipase, or at the level of IP_3_-mediated Ca^2+^ liberation. By directly photoreleasing IP_3_ in the cytosol from a caged precursor (ci-IP_3_)^35^ we found that IP_3_-evoked Ca^2+^ signals in fibroblasts from FXS and TSC subjects were depressed to a similar extent as the ATP-evoked signals. Moreover, by using high-resolution optical imaging (‘optical patch-clamp’)^36^ to resolve Ca^2+^ flux through individual channels, we observed a shortening of the mean open time for all the ASD model cells’ IP_3_R channels, and an apparent decrease in the numbers of discrete release sites. However, the latency to first opening and the mean event amplitudes were similar in all cells. Thus, IP_3_Rs, while carrying no mutations themselves, appear *functionally* altered at the level of single, or small clusters of channels in these three distinct ASD models.

We thus proposed that multiple genetic lesions leading to ASD converge to perturb normal Ca^2+^ signaling, and that depressed function of the IP_3_R Ca^2+^ release channels in the ER play a key ‘hub’ role in the pathogenesis of ASD – one that might serve as a diagnostic biomarker and potential target for novel drug discovery^24^,^34^,^37^. However, that hypothesis was based on studies limited to rare, monogenic forms of ASD. Here, we extend our studies to subjects with typical, sporadic ASD, employing a high-throughput fluorescence reader to assay agonist-evoked Ca^2+^signaling in skin biopsy fibroblasts from a cohort of subjects diagnosed with typical sporadic ASD, in addition to FXS and TSC subjects. We observe that ATP-evoked Ca^2+^ release is depressed in fibroblasts from a majority of sporadic ASD subjects, to an extent similar to that seen in cells from subjects with monogenic forms of ASD.

## Results

### FLIPR assay of ATP-induced Ca^2+^ signaling

**Figure 1:**
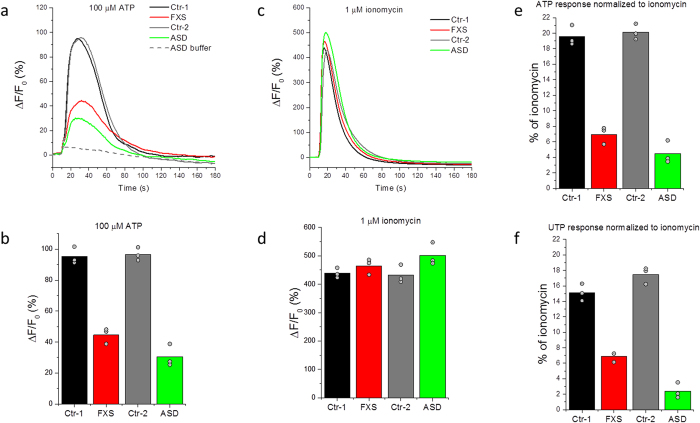
Representative Ca^2+^ responses to extracellular application of purinergic agonists and ionomycin in absence of extracellular Ca^2+^ in fibroblasts from ASD subjects and controls. (**a**) Representative FLIPR traces showing changes in Fluo-8 fluorescence over the basal fluorescence (ΔF/F_0_) in response to extracellular application of 100 μM ATP to fibroblast cell lines from two controls (black; GM03440 and grey; GM02912), one FXS (red; GM09497) and one sporadic ASD subject (green; AU0027-0202). Fluorescence changes ΔF are presented as a % change from the basal fluorescence F_0_. The grey dashed line represents the artifactual fluorescence change resulting from addition of vehicle alone to the ASD cell line. (**b**) Peak amplitudes (ΔF) of Ca^2+^ responses to 100 μM ATP normalized to the basal fluorescence (F_0_) before stimulation in control cell lines (black and grey), FXS (red) and a sporadic ASD line (green). Bar graphs show mean of triplicate measurements after subtracting the artifactual signal resulting from addition of vehicle alone to each corresponding cell line. Data points represent individual triplicate responses. (**c**) Representative FLIPR traces showing changes in fluorescence over the basal (ΔF/F_0_) in response to extracellular application of 1 μM ionomycin to control (black and grey traces), FXS (red) and ASD (green) cell lines. (**d**) Mean peak Ca^2+^ responses (ΔF) to 1 μM ionomycin normalized to the basal fluorescence (F_0_) before stimulation in control cell lines (black and grey), FXS line (red) and an ASD line (green). Bar graphs show mean of triplicate measurements. Data points represent individual triplicate responses. (**e**) Mean peak ATP responses for each cell line from (**b**) expressed as a percentage of the mean ionomycin response from (**d**) in that cell line. (**f**) Mean peak responses evoked by addition of 100 μM UTP to each cell line as a percentage of the of ionomycin response for that cell line.

Intracellular Ca^2+^ release in cultured untransformed primary skin fibroblasts (Table 1) was measured using a high-throughput Fluorometric Imaging Plate Reader (FLIPR). ATP (100 μM final concentration) was applied to activate cell-surface purinergic receptors and induce subsequent IP_3_ production and Ca^2+^ release. Ca^2+^-free extracellular medium supplemented with 1 mM EGTA was used to exclude Ca^2+^ entry across the cell membrane. Representative fluorescence traces illustrating ATP responses in fibroblasts from two neurotypical controls and one with FXS, as well as one from an enrolled subject with typical, sporadic ASD are shown in Fig. 1a. The grey dashed line shows the fluorescence signal change upon addition of vehicle only. We quantified fluorescence signals as a ratio (ΔF/F_0_) of the fluorescence change (ΔF) at each well, after subtracting the change resulting from addition of vehicle alone, relative to the basal fluorescence (F_0_) before stimulation. Figure 1b shows ΔF/F_0_ values (mean of measurements from three plotted replicate wells) from these cell lines in response to 100 μM ATP.

To determine whether differences in intracellular Ca^2+^ store content may have contributed to differences in ATP-evoked Ca^2+^ signals^38^,^39^, we applied 1 μM ionomycin, a specific Ca^2+^ ionophore, to independent wells (Fig. 1c). Ionomycin induces Ca^2+^ release largely from thapsigargin-sensitive ER stores, as a 20-min pre-incubation with 1 μM thapsigargin slowly liberates over 90% of the ionomycin-releasable pool. Thus the ionomycin response can serve as a fast and tractable measure of ER Ca^2+^ content in the Ca^2+^-free extracellular solution. Peak ionomycin response amplitudes, normalized to the basal fluorescence (ΔF/F_0_) from the same ASD and control cells, are shown in Fig. 1d. Ca^2+^ responses to ionomycin were closely similar in ASD and control cell lines, consistent with our previous finding that maximal Ca^2+^ store filling does not differ between monogenic forms of ASD and controls^34^. To account for any differences between individual cell lines in the Ca^2+^ store filling across different 96-well plates and different days, we present all ATP-induced Ca^2+^ signals as a percentage of the ionomycin response evoked in parallel wells on the same plate (Fig. 1e).

Ca^2+^ signals with amplitudes comparable to that evoked by ATP were obtained with 100 μM uridine triphosphate (UTP), an agonist that, like ATP, primarily activates P2Y receptors (Fig. 1f); whereas diphosphates – adenosine diphosphate (ADP) and uridine diphosphate (UDP), and MRS 2365, a selective P2Y1 agonist, all failed to evoke appreciable Ca^2+^ signals at concentrations of 100 μM (Supplemental Figure 1). These data suggest that the receptors being activated by our screening procedure are of the P2Y2, or a combination of P2Y4 and P2Y11, receptor class^40^,^41^, concordant with reported expression of P2Y2 and P2Y4 in human dermal fibroblasts^42^.

### Consistency and reproducibility of the FLIPR assay

We sought to evaluate the extent to which factors including the source, culture initiation, and storage conditions of fibroblast cell lines might affect our Ca^2+^ signaling assay. We enrolled and re-biopsied the same individual who had previously provided cell line GM24529 that was deposited and stored at the Coriell cell biorepository. The resulting cell line (AU0239-0201) was derived from the same individual with a two-year interval between sampling. Cell cultures were initiated, maintained and stored independently either at CART, exactly as for all of the other sporadic ASD samples; or at the Coriell cell biorepository, as were a majority of syndromic ASD cell lines and all of the controls. When run simultaneously on the same plate, both cell lines demonstrated closely similar responses to ATP that were not statistically different when normalized to the ionomycin response (Supplemental Figure 2).

In addition to normalizing ATP-evoked Ca^2+^ signals relative to ionomycin responses, we further sought to mitigate the day-to-day variability typical of high-throughput functional screens such as FLIPR^43^ by expressing ionomycin-normalized responses from each cell line as a percentage of the mean response in triplicate measurements from a “reference” cell line (the control line GM03440) included on the same plate. We chose this cell line as a reference because it demonstrated a robust Ca^2+^ response and had a low passage number (P4) at the time of deposition at Coriell cell biorepository, similar to the passage number (P5) at which CART-derived cells were frozen down. Moreover, this line has been widely used in >20 published studies^44^. Normalizing to the reference cell line reduced day-to-day variability in ATP-evoked Ca^2+^ responses among individual cell lines by an average of 38%, measured using three different cell lines each run on four independent days. For each run a mean value was taken from triplicates and the variability between runs was calculated as the coefficient of variation (CV: standard deviation divided by the mean across the four runs). Respective values of CV for the three lines before and after normalization were: 0.81/0.33 (59% reduction in variability); 1.21/1.08 (11% reduction); 0.44/0.24 (45% reduction).

### ATP-evoked Ca^2+^ signals are depressed in fibroblasts from sporadic ASD subjects

**Figure 2:**
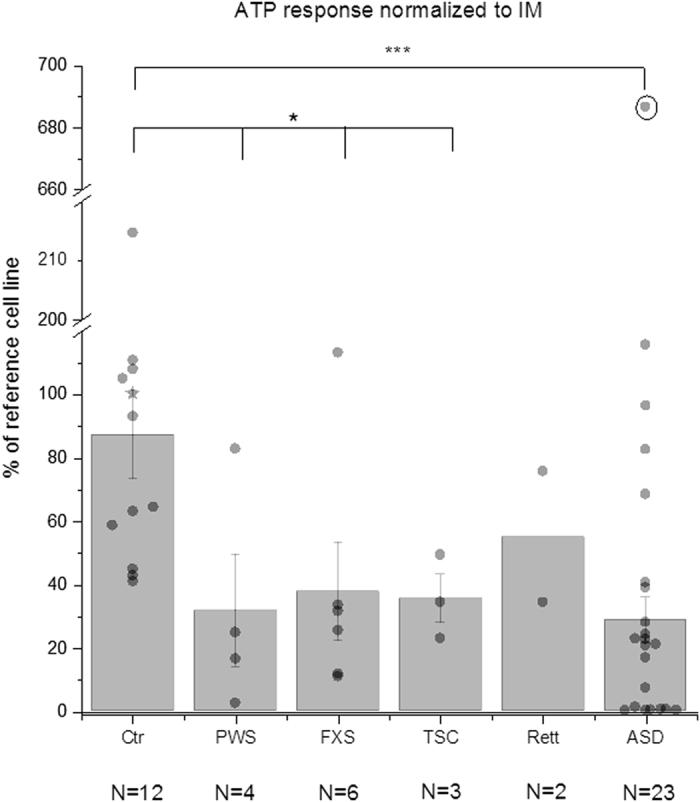
Ca^2+^ response in fibroblasts from subjects with sporadic ASD as well as from controls and those with syndromic ASD. Average Ca^2+^ response in skin fibroblasts from unaffected neurotypical controls (Ctr), Prader-Willi syndrome (PWS), fragile X syndrome (FXS), tuberous sclerosis syndrome 1 and 2 (TSC), Rett syndrome (Rett) and from subjects with sporadic ASD (ASD). N below each cell line represents number of individuals tested. The star symbol represents the reference control cell line (GM03440). Peak Ca^2+^ response (ΔF/F_0_) divided by the peak ionomycin response (ΔF/F_0_) was normalized to the mean value of the reference cell line (GM03440) run on the same FLIPR plate. Bar graphs show mean +/− SEM for each group. Data points represent responses from an individual. Circled data point (AU0237-0201) was excluded from the average and statistics. *p-value < 0.05, ***p < 0.001, Mann-Whitney test.

Monogenic syndromes represent just a small fraction of all ASD cases, with the majority being sporadic, or polygenic (for a review, see ref. 45). To determine whether the IP_3_ signaling defect we observe is a common feature of ASD, or is unique to single-gene mutations, we expanded our observations to fibroblasts from subjects with sporadic forms of ASD as well as two further monogenic syndromes (Prader-Willi syndrome, PWS; and Rett syndrome, Rett). Figure 2 presents ATP-evoked Ca^2+^ responses in fibroblasts from multiple sporadic ASD subjects as well as those from control, PWS, FXS, TSC and Rett syndromes, after normalizing each as a percentage of the Ca^2+^ response to the mean reference cell line included in each plate. Data points (dots) show measurements from individual subjects as means of triplicates; grey bars indicate means of N subjects with error bars showing ±1 SEM. Consistent with our previous report^34^, ATP-evoked Ca^2+^ responses in cells from individuals with FXS and TSC were, on average, substantially reduced (to about 30%) as compared with the mean response in cells from control neurotypical individuals. We further observed a similar reduction in mean responses in cells from individuals with PWS (to 35%) and Rett syndrome (to 62%).

Most importantly, the mean response from cell lines from sporadic ASD subjects was also considerably depressed relative to controls (Fig. 2). A majority of the ASD cells gave very small or no detectable responses, and all control cells gave responses above the mean of the ASD cells (Fig. 2). One ASD cell line consistently gave remarkably discrepant ATP responses, with a mean amplitude close to that evoked by ionomycin (75%) and almost seven times greater than the control average (Fig. 2, circled data point). That subject was shown to carry a chromosomal deletion and is the topic of ongoing investigation. For the present we exclude that cell line from our statistical analysis. The mean response of the remaining 22 cell lines from subjects with sporadic ASD was 28% ± 7% SEM of the reference cell line as compared to 87% ± 14% for the controls; a reduction to about 31%. However, despite the smaller average response, Ca^2+^ signal amplitudes among the cell lines from subjects with sporadic ASD displayed a much wider spread than the controls, and six of the ASD subjects had cell responses that overlapped those of the controls. This high variability and skewed, non-normal Gaussian distribution points to considerable heterogeneity of Ca^2+^ signaling among the cohort of sporadic ASD subjects.

### ROC curves discriminate between ASD subjects and controls

**Figure 3:**
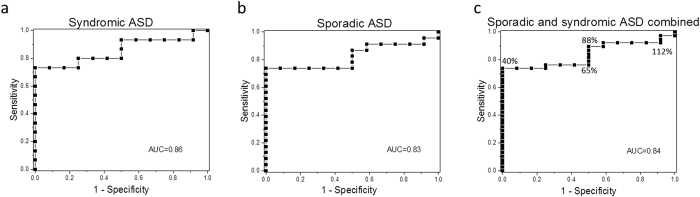
Receiver operating characteristic (ROC) curves for ATP-evoked Ca^2+^ signaling in ASD. (**a**) ROC curve results for syndromic ASD cell lines (N = 15) and unaffected neurotypical controls (N = 12). Sensitivity (the true-positive rate) was plotted against (1-specificity) (the false-positive rate) for each value of Ca^2+^ signaling response normalized to a reference control cell line (the data are the same as in Fig. 2). Only subjects with known identified genetic syndromes co-morbid with ASD (FXS (N = 6), Rett (N = 2), PWS (N = 4), TSC (N = 3)) were used to generate the curve. Area under the curve (AUC) is shown in each graph. (**b**) ROC curve results for sporadic ASD subjects (N = 23) and unaffected neurotypical controls (N = 12). (**c**) ROC results for Ca^2+^ signaling in sporadic and syndromic ASD cohorts combined from (**a**) and (**b**). Numbers in % reflect Ca^2+^ signaling cutoff values (presented as % of the reference cell line) to illustrate how different threshold values influence specificity and sensitivity of the ROC curve.

To assess the robustness of the difference between cell lines from subjects with sporadic ASD and controls, we generated receiver operating characteristic (ROC) curves (Fig. 3); a metric that is widely used to evaluate parameters to separate affected from unaffected individuals for diagnostic purposes^46^,^47^. The ROC curve expresses the accuracy of a test in terms of two measures – sensitivity and specificity – in this case comparing the Ca^2+^ signaling assay against the Autism Diagnostic Observation Schedule (ADOS) assessment as a ‘gold standard’ for diagnosis of ASD. Thus, sensitivity refers to the proportion of subjects who are correctly identified by the assay as having ASD (true positive): a highly sensitive test best assures that affected people will be identified. Specificity refers to the true-negative rate: here, the proportion of subjects without ASD who are correctly identified as not having the condition. At any given Ca^2+^ signaling value, the sensitivity and the specificity are calculated from a ratio of people who are disease positive (true positive) or disease negative (false positive) at that threshold. For example, it is apparent from Fig. 2 that a low Ca^2+^ signal cutoff value would exclusively capture subjects with ASD, but would not capture all of the affected subjects. As the signaling cutoff is increased, more ASD subjects are captured, so the *sensitivity* increases, but the number of unaffected controls captured increases, thereby decreasing the *specificity*. The ROC curve therefore essentially describes the compromise between sensitivity and specificity of a test at varying threshold cutoff values.

After sorting all subjects by their Ca^2+^ signaling normalized to the reference cell line (as was done for Fig. 2), we generated an ROC curve by plotting sensitivity (true positive rate) against 1-specificity (false positive rate) at each test value for individuals with syndromic ASD (FXS, TSC1 and TSC2, Rett and PWS) as shown in Fig. 3a. The area under the ROC curve (AUC) is a useful tool to compare the utility of a biomarker. It represents the overall probability that the correct diagnostic status (ASD *vs* unaffected in our case) will be accurately identified in a randomly chosen individual, with an AUC of 1 having a perfect predictive value and 0.5 being no better than random. The ROC generated for syndromic ASD resulted in a robust AUC of 0.86, a value considered an excellent discriminant in predicting disease status^48^.

Notably, our cohort of subjects with sporadic ASD yielded an ROC curve (Fig. 3b) closely resembling that of syndromic ASD, with a similar AUC of 0.83. An ROC curve pooling individuals with both syndromic and sporadic ASD (Fig. 3c) yielded an AUC of 0.84. The similarity of our findings between sporadic ASD subjects and those with diverse monogenic syndromes suggests a common underlying signaling deficit across different forms of ASD. Using a cutoff value at 40% of the “reference” cell-normalized ATP-evoked Ca^2+^ signal achieved 73% sensitivity and 100% specificity for discriminating between pooled ASD subjects and controls, irrespective of their genetic background (Fig. 3c). More work, including replication studies with larger cohort sizes, would be needed to establish a reliable diagnostic test, but our findings indicate that Ca^2+^ signaling may be a new promising biomarker target in ASD.

## Discussion

ASD is a broad, umbrella diagnosis for a heterogeneous group of conditions encompassing several neurodevelopmental problems, along with many phenotypes and co-morbidities. Biomarkers of ASD have long been sought in the hopes that they might improve outcomes for these patients and their families. We had previously demonstrated that patient-derived fibroblasts from three monogenic models of ASD (FSX, TSC1 and TSC2) display depressed Ca^2+^ release evoked by purinergic receptor activation of IP_3_ signaling, and proposed that dysregulation of IP_3_ signaling constitutes a nexus where genes altered in ASD converge to exert their deleterious effect^34^. Here, we extend those findings to reveal a corresponding deficit in IP_3_-mediated Ca^2+^ release in cells from subjects with sporadic ASD, where each subject likely carries a unique sampling of genetic risk alleles. By using a high throughput assay to measure Ca^2+^ signals evoked by ATP in fibroblasts from subjects with ASD and controls we are able to derive an ROC curve that can discriminate subjects with ASD from unaffected controls with high sensitivity and specificity. Notably, this approach identifies subjects with highly heterogeneous sporadic forms of ASD as well as a spectrum of homogeneous monogenic syndromes caused by “major effect” mutations, and does so similarly well with both, pointing to a common signaling defect in the ubiquitous IP_3_-mediated Ca^2+^ signaling pathway. Even though the number of subjects used in this study was modest and the results need to be replicated with larger cohorts of ASD subjects and neurotypical controls, it serves as a proof of principle for the prospective utility of such testing.

Fibroblasts are primary, untransformed cells that are readily obtained by skin biopsy. A patient-derived, cell-based assay such as we describe here has potential as a biomarker for early detection of children susceptible to ASD, before behavioral symptoms appear and when an earlier intervention has a better chance of improving outcome^3^,^4^. Although several blood-based biomarkers with high specificity and sensitivity have been proposed for ASD^49^,^50^,^51^, they are not currently suitable for high-throughput screening, and may be subject to alteration due to medication regimen, diet, lifestyle changes or other variables that would potentially complicate the read-out.

The current practice of testing new ASD treatments in biologically and behaviorally heterogeneous populations of ASD subjects is widely acknowledged to impede the identification of new drugs that would be effective in only a specific subgroup of “responders”^52^,^53^. Therefore, there is hope that a set of biomarkers could independently stratify patient populations into distinct, biologically meaningful endophenotypes^54^ to enable more robust clinical trials. Although limited to a modest cohort of subjects, our results already hint at such a stratification of Ca^2+^ signal amplitudes among sporadic ASD subjects, which exhibit a much greater spread of signaling responses than controls, with a majority giving almost no response whereas others exhibit signals overlapping the control range.

In addition to their potential utility as a biomarker and diagnostic tool, functional assays based on patient-derived cells have potential for high-throughput *in vitro* screening for novel candidate drugs. This is particularly relevant in light of the present inadequacy of animal models of ASD, a critical concern given the failure of multiple clinical trials that were predicated upon animal data^52^,^55^. Indeed, the promise of cell-based approaches to ASD is underscored by the now well-established utility of going directly to human disease cells for drug discovery in instances where animal models are lacking; as was the case for cystic fibrosis where novel therapeutics were identified based on high throughput screening of patient cells for correction of a patient’s cellular biomarker phenotype^56^,^57^,^58^.

In summary, the results presented here have significant mechanistic and translational relevance that support the involvement of Ca^2+^ signaling disruptions in ASD. Although independent replications and refinements with the use of much larger cohorts will be required, our study opens the prospect that a skin biopsy sample could become a functional cellular diagnostic and surrogate clinical trial outcome end-point measure, much as long has been the case for neurogenetic encephalopathies caused by defects in mitochondria, lysosomes and peroxisomes^59^,^60^,^61^,^62^. Using this cell-based assay, novel or repurposed candidate drugs could be rapidly screened to evaluate their efficacy on a subject’s cells prior to their enrollment in a clinical trial, hopefully improving the prospects for autistic children and their families.

## Methods

### Materials

Fluo-8 AM was purchased from AAT Bioquest, diluted in DMSO (Sigma D2650) to a stock concentration of 2 mM and frozen as 25 μl aliquots until needed. On the day of the experiment the Fluo-8 AM solution was thawed and diluted with an equal volume of 20% Pluronic F-127 (Molecular Probes, P6867) prepared in DMSO. Adenosine triphosphate (ATP), adenosine diphosphate (ADP), uridine triphosphate (UTP) and uridine diphosphate (UDP) were purchased from Sigma Aldrich, diluted in water to a stock concentration of 100 mM and frozen as 50 μl aliquots until needed. MRS 2365 (supplied pre-dissolved at a concentration of 10 mM) was purchased from Tocris. Ionomycin was purchased from Life Technologies, diluted in DMSO to 1 mM and frozen as 10 μl aliquots until needed.

### Subject fibroblast cell lines

All methods were carried out in accordance with relevant guidelines and regulations, and all experimental protocols were approved by UCI Institutional Review Board (IRB) review. Skin fibroblast cultures were obtained from sporadic ASD subjects enrolled into the UCI Center for Autism Research and Translation (CART). All CART-derived cell lines reported here were from subjects who were referred with a clinical diagnosis of ASD. Three such subjects had Prader-Willi syndrome and are classified as such. CART subjects underwent a full day of testing to develop their deep phenotype, including skin biopsy, all obtained with informed consent and assent. Age-appropriate research-grade ADOS and IQ tests were administered, followed by a set of high-density EEG studies, a sleep deprivation study and preparation for a follow-up at home 5-day sleep study with accelerometers and app-assisted parent sleep and behavior logging. Metabolomic studies of blood, urine, saliva and volatile metabolites in breath were obtained, as well as blood from the subject and family members for whole genome sequencing. Only those subjects with validated ADOS scores in the “Autism” or the “Autism Spectrum Disorder” ranges were selected for study (Table 1). Fibroblast cell lines were established from punch skin biopsy (2–3 mm) explants and frozen at passage 5 in liquid nitrogen for long-term storage.

Primary, untransformed skin biopsy fibroblast cultures from neurotypical controls and monogenic forms of ASD (fragile X syndrome, tuberous sclerosis, Rett, and one with Prader-Willi syndrome) were obtained from Coriell cell biorepository.

Fibroblast were cultured in Dulbecco’s Modified Eagle’s Media (Gibco, 11965-092) supplemented with 20% (v/v) fetal bovine serum without antibiotics at 37 °C in a humidified incubator gassed with 95% air and 5% CO_2_, and used for up to 15 passages. Cells were studied at passages 10–15. For Ca^2+^ signaling studies, cells were detached with Ca^2+^- and Mg^2+^-free 0.25% trypsin-EDTA (Life Technologies), harvested in normal growth media and sub-cultured on FLIPR 96 well plates for 2 days to provide standardized conditions prior to imaging studies.

### High-throughput Ca^2+^ imaging

Skin fibroblasts were seeded in clear-bottom black 96-well plates (Greiner Bio One T-3026-16) at 1 × 10^4^ cells per well and grown to confluency. On the day of the experiment, cells were loaded by incubation with 2 μM of the membrane-permeant Ca^2+^ indicator Fluo-8 AM^63^ in standard buffer solution (130 mM NaCl, 2 mM CaCl_2_, 5 mM KCl, 10 mM glucose, 0.45 mM KH_2_PO_4_, 0.4 mM Na_2_HPO_4_, 8 mM MgSO_4_, 4.2 mM NaHCO_3_, 20 mM HEPES and 10 μM probenecid, pH 7.4 at the room temperature) with 0.1% fetal bovine serum for 1 h at 37 °C, then washed with a Ca^2+^ -free HBSS solution (120 mM NaCl, 4 mM KCl, 2 mM MgCl_2_, 10 mM glucose, 10 mM HEPES, 1 mM EGTA, pH 7.4 at the room temperature) once. The solution was replaced with 100 μl of fresh Ca^2+^-free HBSS solution in each well and cells were allowed to equilibrate for 5 minutes prior to assay with a Fluorometric Imaging Plate Reader (FLIPR; Molecular Devices, Sunnyvale, CA). A basal read of fluorescence in each well (470–495 nm excitation and 515–575 nm emission, expressed in arbitrary units; AU) was read for 2 seconds at 0.4 s exposure time. Next, 100 μl of 2x ATP (to 100 μM final concentration) or 100 μl of 2x ionomycin (to 1 μM final concentration) in Ca^2+^ -free HBSS was added to a given well. Only a single recording was obtained from each well. Ionomycin-induced fluorescence changes from wells without prior addition of ATP were used to normalize ATP-evoked responses. Recordings were performed in triplicate. Each experiment was repeated on at least two independent days.

### Data processing and analysis

The peak change in fluorescence amplitude (ΔF) in each well was normalized to the basal fluorescence of that well before stimulation (F_0_) after subtraction of the camera black offset level. Mean ATP responses from triplicate wells were further normalized to the triplicate-average ΔF/F_0_ of the ionomycin response from each corresponding cell line from the same plate to express the ATP-releasable Ca^2+^ pool as a proportion of the total cellular Ca^2+^ store content. To mitigate plate-to-plate and day-to-day variability, mean ATP/ionomycin responses for each cell line from individual wells were divided by the ATP/ionomycin ratio of a reference cell line (GM03440) (mean of triplicates) included on each plate. All data are presented as mean ± 1 SEM Mann-Whitney test was used to determine statistical significance of the findings.

OriginPro 2015 (Origin Lab Corp., Northampton, Massachusetts) was used for data analysis and graph plotting.

## Additional Information

**How to cite this article**: Schmunk, G. *et al*. High-throughput screen detects calcium signaling dysfunction in typical sporadic autism spectrum disorder. *Sci. Rep.* **7**, 40740; doi: 10.1038/srep40740 (2017).

**Publisher's note:** Springer Nature remains neutral with regard to jurisdictional claims in published maps and institutional affiliations.

## Supplementary Material

Supplementary Information

## Figures and Tables

**Table 1:** Skin fibroblast information for ASD subjects and controls.

*Monogenic ASD*						*Sporadic ASD*						*Control*					
ID	Sex	Age	Ethnicity	Status	Ca2^+^ signal	ID	Sex	Age	Ethnicity	Status	Ca2^+^ signal	ID	Sex	Age	Ethnicity	Status	Ca2^+^ signal
GM09497	M	28	Caucasian	FXS	33.4	AU0001-0201	M	29	Caucasian	Autism	82.5	GM00498	M	3	N/A	Healthy	104.8
GM05848	M	4	Caucasian	FXS	10.8	AU0027-0201	F	24	Caucasian	Autism	115.5	GM01863	M	46	Caucasian	Healthy	58.5
GM05185	M	26	Caucasian	FXS	11.7	AU0027-0202	M	21	Caucasian	Autism	22.9	GM02185	M	36	Caucasian	Healthy	107.8
GM05131	M	3	Caucasian	FXS	25.3	AU0078-0202	F	36	Caucasian	Autism	96.3	GM02912	M	26	Caucasian	Healthy	64.3
GM04026	M	35	Caucasian	FXS	31.4	AU0120-0202	M	15	Asian	Autism	24.2	GM03440	M	20	Causasian	Healthy	100.0
GM04024	M	29	Black	FXS	113.0	AU0197-0201	M	17	Hispanic	PDD-NOS	38.9	GM04505	F	20	N/A	Healthy	110.6
GM21890	M	19	N/A	PWS	82.7	AU0197-0202	F	14	Hispanic	PDD-NOS	22.6	GM05659	M	1	Caucasian	Healthy	40.8
GM16548	F	5	Caucasian	RETT	34.2	AU0236-0203	F	12	Caucasian	Autism Spectrum	7.2	GM07492	M	17	Caucasian	Healthy	44.7
GM07982	F	25	Caucasian	RETT	75.6	AU0237-0201	F	13	Caucasian	Autism	686.6	GM07753	M	17	N/A	Healthy	214.6
GM06149	M	17	Caucasian	TSC1	49.2	AU0239-0201	F	16	Caucasian	Autism	21.3	GM08399	F	19	N/A	Healthy	62.9
GM06148	M	43	Caucasian	TSC1	34.3	AU0239-0203	F	6	Caucasian	Autism Spectrum	0.5	GM23973	M	19	Caucasian	Healthy	42.7
GM06121	M	22	Caucasian	TSC2	22.8	AU0243-0201	M	2	Caucasian	Autism	0.5	GM23976	M	22	Caucasian	Healthy	93.0
AU0240-0203	M	12	Caucasian	PWS	2.4	AU0245-0201	F	20	Caucasian	Autism	68.4						
AU0244-0201	M	19	Caucasian	PWS	24.8	AU0245-0202	F	18	Caucasian	Autism Spectrum	28.0						
AU0250-0202	M	11	Caucasian	PWS	16.4	AU0249-0202	F	10	Caucasian	Autism	16.7						
						AU0251-0202	M	8	Hispanic	Autism	40.6						
						AU0251-0203	M	5	Hispanic	Autism	22.8						
						AU0252-0201	M	4	Asian	Autism	20.6						
						AU0254-0201	M	5	Hispanic	Autism Spectrum	0.3						
						AU0254-0202	F	3	Hispanic	Autism	0.3						
						AU0256-0201	M	5	Caucasian	Autism	0.2						
						AU0256-0202	M	3	Caucasian	Autism	0.1						
						AU0257-0202	M	3	Asian	Autism	1.2						

Controls are defined as apparently healthy individuals without any known neurodevelopmental disorders (Coriell cell biorepository). Cell lines starting with “GM#” were purchased from the Coriell cell biorepository; cell lines starting with “AU#” were established by UCI CART. The ASD diagnosis was established based on the results of administered appropriate version of ADOS. PDD-NOS = Pervasive Developmental Disorder-Not Otherwise Specified.

Ca^2+^ signals are presented as percentage of the reference cell line (GM03440).
