# Supplementary material for: High-throughput screen detects calcium signaling dysfunction in typical sporadic autism spectrum disorder
Source: Sci Rep. 2017 Feb 1;7:40740. doi: 10.1038/srep40740 (PMC5286408; doi:10.1038/srep40740)
Supplement: Supplementary Information [file srep40740-s1.pdf]

Supplementary Information

Title:

"High-throughput screen detects calcium signaling dysfunction in typical sporadic autism spectrum disorder"

Authors:

Galina Schmunk*[1,4], Rachel L. Nguyen[4], David L. Ferguson[1,4], Kenny Kumar[4], Ian Parker[1,3] & J. Jay Gargus[1,2,4]

Affiliations:

[1]Department of Physiology and Biophysics, School of Medicine, University of California, Irvine, California, USA.
[2]Division of Human Genetics & Genomics, Department of Pediatrics, School of Medicine, University of California, Irvine, California, USA.
[3]Department of Neurobiology and Behavior, School of Biological Sciences, University of California, Irvine, California, USA.
[4]Center for Autism Research and Translation, University of California, Irvine, California, USA.

***Correspondence:**

Galina Schmunk
1146 McGaugh Hall
University of California, Irvine
Irvine, CA 92697-3940 USA
gschmunk@uci.edu

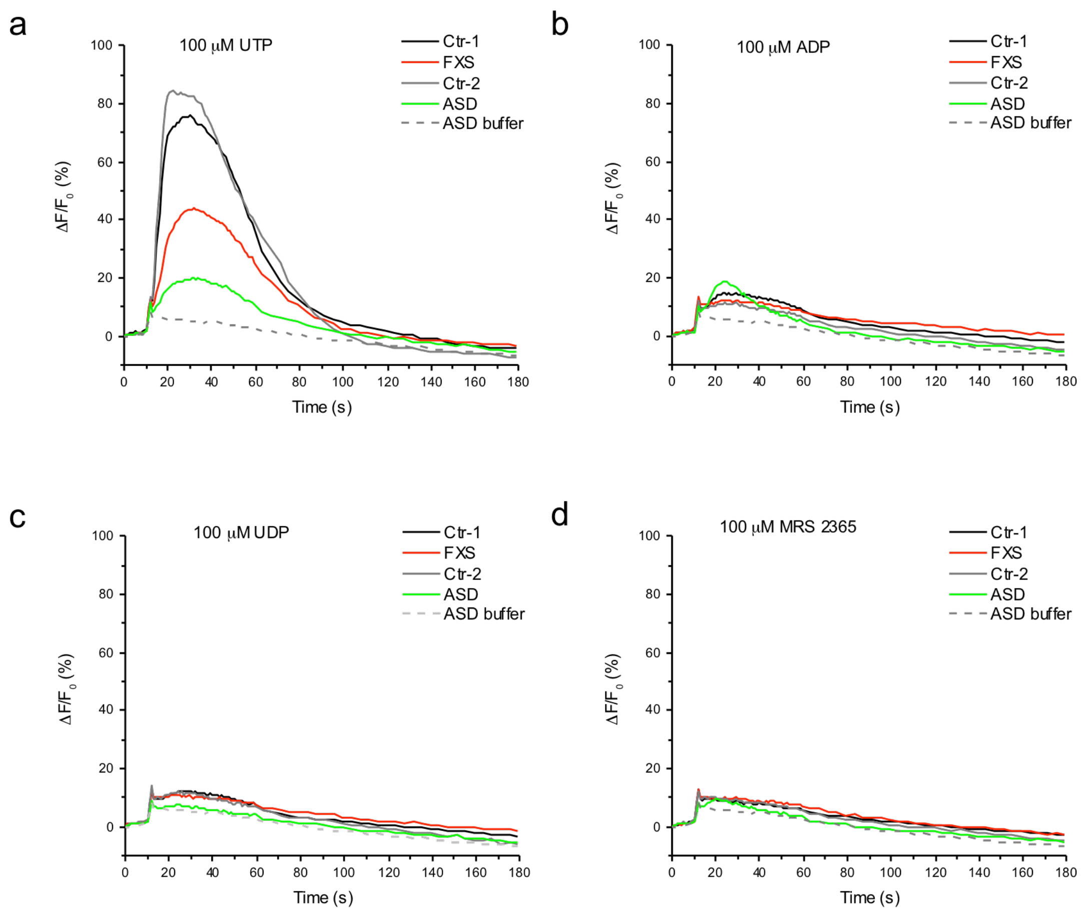

Supplemental Figure 1. Representative Ca^{2+} responses to extracellular application of different purinergic receptor agonists in absence of extracellular Ca^{2+} in fibroblasts from control and ASD patients.

(a) Representative FLIPR traces showing change in fluorescence over the basal ($\Delta F/F_0$) in response to extracellular application of 100 μM UTP in control (black and grey traces), FXS (red) and sporadic ASD (green) cells loaded with the Ca^{2+} indicator Fluo-8. Grey dashed line represents fluorescence response of the ASD line to a vehicle addition alone. Ca^{2+}-free buffer contains 1 mM EGTA. **(b)** Representative FLIPR traces showing insignificant response to extracellular application of 100 μM ADP. Color legend is the same as in (**a**). **(c)** Representative FLIPR traces showing minimal response to extracellular application of 100 μM UDP. **(d)** Representative FLIPR traces showing response to extracellular application of 100 μM MRS 2365.

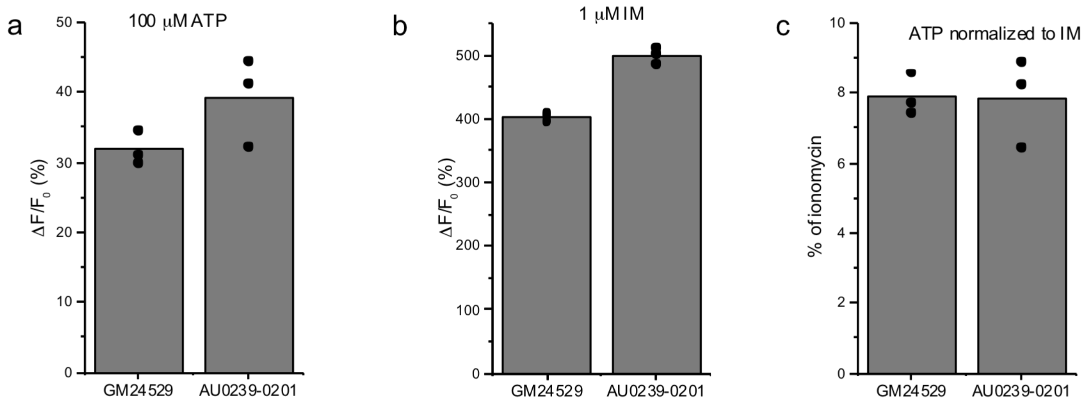

Supplemental Figure 2. Ca^{2+} response in two different fibroblast cell lines derived from the same patient.

(a) Peak amplitude (ΔF) Ca^{2+} response to 100 μM ATP normalized to the basal fluorescence (F_0) before stimulation. The data were calculated by subtracting vehicle addition peak response from peak trace value of each corresponding cell line. Bar graphs show mean of triplicate measurements. The cell line GM24529 was established by Coriell cell biorepository (Camden, New Jersey). The same patient was re-biopsied at CART, UC Irvine, and a cell culture (AU0239-0201) was established from an explant. Both cell lines were thawed from a liquid nitrogen long-term storage, passaged and plated for high-throughput Ca^{2+} signaling in parallel. **(b)** Peak amplitude Ca^{2+} response to 1 μM ionomycin normalized to the basal fluorescence before stimulation. **(c)** Peak ATP response for each cell line from (**a**) normalized to that of ionomycin response from (**b**). Bar graphs show mean of triplicate measurements from individual wells. Data points represent individual triplicate responses.
